# TRAIL (TNF-related apoptosis-inducing ligand) inhibits human adipocyte differentiation *via* caspase-mediated downregulation of adipogenic transcription factors

**DOI:** 10.1038/cddis.2016.286

**Published:** 2016-10-13

**Authors:** Verena Zoller, Jan-Bernd Funcke, Michaela Keuper, Muad Abd El Hay, Klaus-Michael Debatin, Martin Wabitsch, Pamela Fischer-Posovszky

**Affiliations:** 1 Division of Pediatric Endocrinology and Diabetes, Department of Pediatric and Adolescent Medicine, University Medical Center Ulm, Ulm, Germany; 2 Department of Pediatric and Adolescent Medicine, University Medical Center Ulm, Ulm, Germany

## Abstract

Tumor necrosis factor-*α* (TNF*α)* and other ligands of the TNF superfamily are potent regulators of adipose tissue metabolism and play a crucial role in the obesity-induced inflammation of adipose tissue. Adipose tissue expression levels of TRAIL (TNF-related apoptosis-inducing ligand) and its receptor were shown to be upregulated by overfeeding and decreased by fasting in mice. In the present study we aimed to elucidate the impact of TRAIL on adipogenesis. To this end, human Simpson-Golabi-Behmel syndrome (SGBS) preadipocytes as well as stromal-vascular cells isolated from human white adipose tissue were used as model systems. Human recombinant TRAIL inhibited adipogenic differentiation in a dose-dependent manner. It activated the cleavage of caspase-8 and -3, which in turn resulted in a downregulation of the key adipogenic transcription factors C/EBP*α*, C/EBP*δ*, and PPAR*γ*. The effect was completely blocked by pharmacological or genetic inhibition of caspases. Taken together we discovered a so far unrecognized function of TRAIL in the regulation of adipogenesis. Targeting the TRAIL/TRAIL receptor system might provide a novel strategy to interfere with adipose tissue homeostasis.

Tumor necrosis factor-related apoptosis-inducing ligand (TRAIL) is a member of the tumor necrosis factor (TNF) superfamily.^1, 2, 3^ TRAIL interacts with four transmembrane receptors (TRAIL-R1 to TRAIL-R4) and with the soluble receptor osteoprotegerin, all belonging to the TNF receptor (TNFR) superfamily.^2^ Only TRAIL-R1 and TRAIL-R2 are capable of transmitting a TRAIL signal from outside to inside of a cell.^2^ In the canonical pathway, binding of TRAIL to TRAIL-R1 or -R2 leads to the assembly of the death-inducing signaling complex (DISC, primary complex) consisting of receptor oligomers, Fas-associated death domain (FADD) as an adaptor molecule, the initiator caspases-8 or -10, and the regulatory protein cellular FLICE-like inhibitory protein (cFLIP). This assembly triggers activation of caspase-8 by proximity-induced dimerization and subsequent self-cleavage, which induces further downstream events and finally apoptosis.^1, 4, 5^ Besides this classical route of signal transduction, TRAIL can activate alternative, non-canonical pathways. This involves the release of a secondary complex consisting of FADD, cFLIP, caspase-8 or -10, and in addition the adaptor protein TNF receptor type 1-associated death domain (TRADD), receptor-interacting protein kinase 1 (RIPK1), and TNF receptor-associated factor 2 (TRAF2) from the DISC.^6, 7, 8^ The secondary complex mediates activation of several intracellular signaling cascades including NF*κ*B, Akt, and mitogen-activated protein kinases (MAPK, i.e. p38, JNK, ERK1/2).^8^

TRAIL was first discovered as an apoptosis-inducing factor. It became famous as a potential anticancer agent because it seemed to selectively induce apoptosis in cancer cells.^3, 9^ Although preclinical studies were promising, clinical trials applying either human recombinant TRAIL or agonistic antibodies revealed only a limited therapeutic benefit (summary of clinical studies in Lemke *et al.*^9^). Therefore, current efforts focus on the identification of compounds, which potently and selectively sensitize cancer cells to TRAIL-induced apoptosis.^9^

Recently, TRAIL is increasingly studied in the context of nonmalignant diseases, specifically metabolic diseases such as obesity, type 2 diabetes mellitus, and cardiovascular disease.^10, 11^ Choi *et al.*^12^ demonstrated that circulating TRAIL levels correlate positively with the body mass index (BMI) and serum lipid levels. Accordingly, Brombo *et al.*^13^ found a significant correlation of serum TRAIL with waist circumference and with LDL-cholesterol. In an experimental setup of physical inactivity, serum TRAIL levels increased with overfeeding, but decreased upon caloric restriction in healthy subjects.^14^ This is in line with our finding that the adipose tissue mRNA expression of TRAIL robustly decreased upon fasting in C57/BL6 mice and rapidly increased again after re-feeding.^15^

Despite TRAIL receptor expression, both preadipocytes and adipocytes are resistant to TRAIL-induced apoptosis.^16, 17^ Instead, TRAIL exerts several non-apoptotic functions in the context of adipose tissue. For example, it regulates insulin-stimulated metabolic processes such as glucose uptake in a caspase-dependent manner.^15^ Moreover, TRAIL was identified as a potent proliferative factor in preadipocytes.^18^ Not only the pool of precursor cells but also the number of mature adipocytes represent important regulators of adipose tissue homeostasis.^19, 20^ Therefore, the aim of the current study was to elucidate the impact of TRAIL on adipogenic differentiation.

## Results

### TRAIL inhibits adipogenic differentiation of human SGBS cells and human primary stromal-vascular cells

To study the effect of TRAIL on adipogenic differentiation, we used human Simpson-Golabi-Behmel syndrome (SGBS) cells,^21^ which express both TRAIL-R1 and TRAIL-R2, but show a very low sensitivity to TRAIL-induced apoptosis.^15, 17, 18^

**Figure 1:**
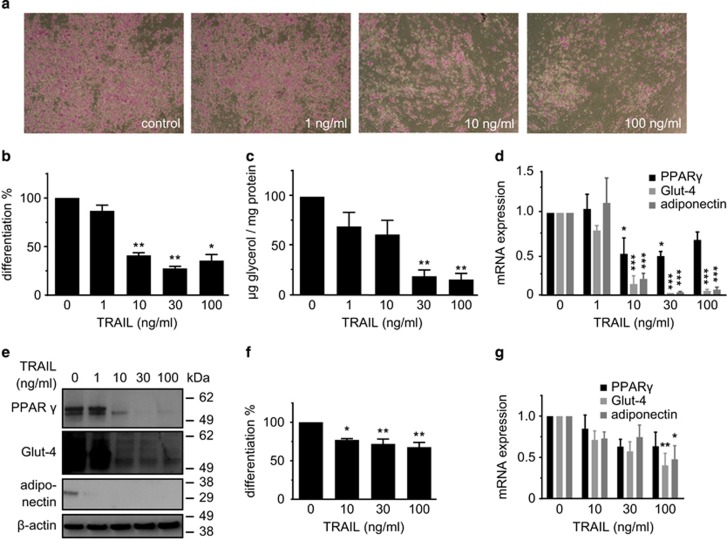
TRAIL inhibits adipogenic differentiation in human SGBS cells. Human SGBS cells were treated with different doses of TRAIL (1–100 ng/ml) during the first 4 days of adipogenic differentiation. Analyses were performed on day 10 of differentiation. (**a**) Representative photomicrographs of cultures stained with the lipophilic dye Oil Red O, magnification × 40. (**b**) The rate of adipogenic differentiation was determined by cell counting. Displayed are the means and S.E.M. of 3–5 independent experiments. (**c**) The cellular triglyceride content was measured and normalized to protein content. Displayed are the means and S.E.M. of four independent experiments. (**d**) RNA was isolated and adipocyte marker gene expression (PPAR*γ*, Glut-4, adiponectin) was determined by qPCR. The mRNA levels were normalized to HPRT. Displayed are the means and S.E.M. of three independent experiments. (**e**) Results were confirmed on the protein level by western blot analysis. *β*-Actin was used as a loading control. The positions of the molecular weight markers (kDa) are indicated. One representative out of three experiments performed is presented. (**f**) Human primary stromal-vascular cells were isolated from subcutaneous adipose tissue samples of five patients. The cells were incubated with different doses of TRAIL (1–100 ng/ml). The rate of adipogenic differentiation was determined on day 10 of differentiation by cell counting. (**g**) RNA was isolated and adipocyte marker gene expression (PPAR*γ*, Glut-4, adiponectin) was determined by qPCR. The mRNA levels were normalized to the gene HPRT. One-way ANOVA and Turkey's multiple comparison were used to test for statistical significance in (**b**–**d**, **f** and **g**). *P<0.05; **P<0.01; ***P<0.001, vehicle *versus* TRAIL

We incubated SGBS cells with increasing doses of TRAIL during the first 4 days of the adipogenesis protocol. Already at a concentration of 10 ng/ml a clear decrease in intracellular lipid droplet formation was visible when cells were stained with the lipophilic dye Oil Red O on day 10 (Figure 1a). Morphological evaluation revealed a reduced rate of adipogenic differentiation (Figure 1b). Likewise, the triglyceride content was significantly reduced in a dose-dependent manner (Figure 1c). These changes were accompanied by a robust downregulation of the adipogenic marker genes PPAR*γ*, Glut-4, and adiponectin on the mRNA (Figure 1d) and the protein level (Figure 1e). Importantly, TRAIL also inhibited adipogenic differentiation in human primary stromal-vascular cells isolated from subcutaneous adipose tissue samples of five healthy donors (Figures 1f and g).

### The antiadipogenic effect of TRAIL is mediated *via* TRAIL-R2

**Figure 2:**
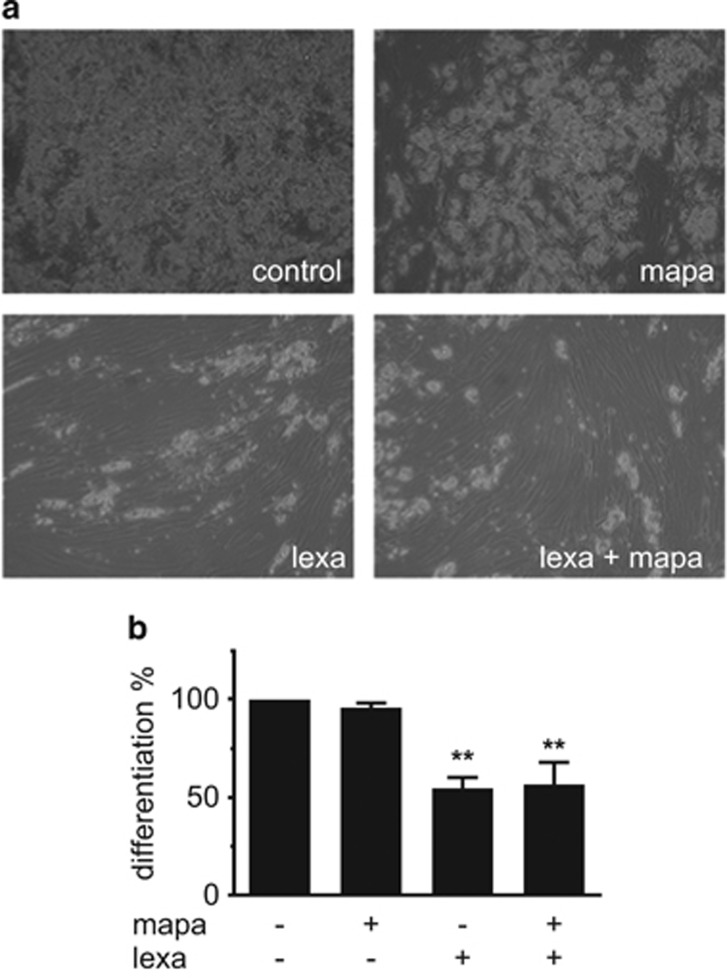
Effect of TRAIL on adipogenic differentiation is mediated by TRAIL-R2. SGBS cells were treated with 10 *μ*g/ml of specific agonistic antibodies for either TRAIL-R1 (mapatumumab, mapa) or TRAIL-R2 (lexatumumab, lexa) or with both together during the first 4 days of adipogenic differentiation. Analyses were performed on day 10 of differentiation. (**a**) Representative photomicrographs of treated cultures, magnification × 40. (**b**) The rate of adipogenic differentiation was determined by cell counting. Displayed are the means and S.E.M. of three independent experiments. One-way ANOVA and Turkey's multiple comparison were used to test for statistical significance. **P<0.01, vehicle *versus* mapa and/or lexa

To elucidate which TRAIL receptor mediates this antiadipogenic effect, we used agonistic antibodies specific for either TRAIL-R1 (mapatumumab) or TRAIL-R2 (lexatumumab). These compounds are currently tested for their anticancer activity in phase I/II studies.^22, 23^ Adipogenic differentiation was not affected when TRAIL-R1 was stimulated with mapatumumab. However, targeting TRAIL-R2 with lexatumumab resulted in a clear inhibition of adipogenesis (Figures 2a and b), indicating that TRAIL-R2 is responsible for mediating the antiadipogenic effect of TRAIL.

### Non-canonical signaling is not involved in mediating the antiadipogenic effect of TRAIL

**Figure 3:**
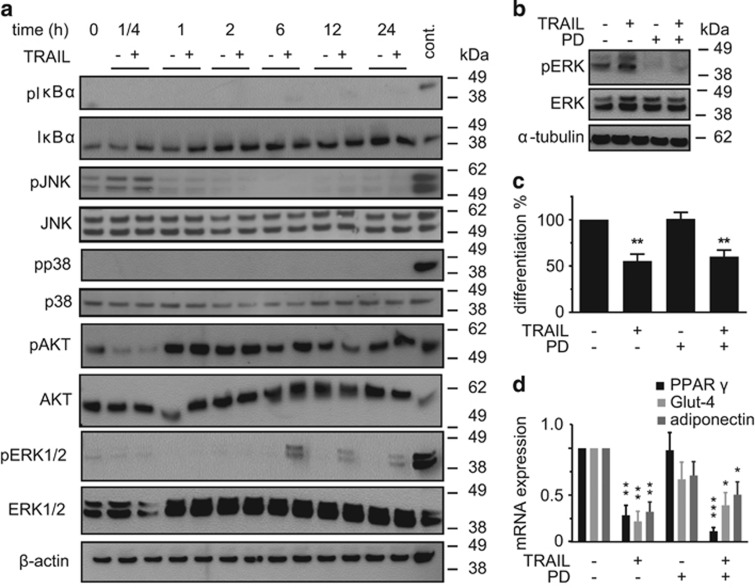
TRAIL induces the activation of ERK1/2, but ERK1/2 is not involved in the effect of TRAIL. (**a**) SGBS cells were treated with TRAIL (30 ng/ml) for different time points (15 min, 1, 2, 6, 12, and 24 h). Protein was isolated and the phosphorylation of I*κ*B*α*, JNK, p38, Akt, and ERK1/2 was analyzed by western blot. *β*-Actin was used as a loading control. The positions of the molecular weight markers (kDa) are indicated. One representative out of three experiments performed is presented. (**b**–**d**) Human SGBS cells were treated with TRAIL (30 ng/ml) during the first 4 days of adipogenic differentiation in the absence or presence of the MEK1/2 inhibitor PD98059 (100 *μ*M). (**b**) The inhibition of ERK1/2 phosphorylation by PD98059 was confirmed by western blot. Here, cells were stimulated for 6 h. One representative out of three experiments performed is presented. (**c**) The rate of adipogenic differentiation was determined by cell counting on day 10 of differentiation. Displayed are the means and S.E.M. of three independent experiments. (**d**) RNA was isolated and adipocyte marker gene expression (PPAR*γ*, Glut-4, adiponectin) was determined by qPCR. The mRNA levels were normalized to the gene HPRT. Displayed are the means and S.E.M. of three independent experiments. One-way ANOVA and Turkey's multiple comparison were used to test for statistical significance in (**c** and **d**). *P<0.05; **P<0.01; ***P<0.001, vehicle *versus* TRAIL and/or PD

TRAIL mediates many of its non-apoptotic effects by non-canonical signaling routes activating NF*κ*B or kinases such as JNK, p38, Akt, and ERK1/2. We first studied NF*κ*B, because this pathway is known to conduct many of the TNF*α*-induced effects on adipose tissue.^8^ There was no phosphorylation of IkB*α* with TRAIL, while stimulation with macrophage-conditioned medium, which was performed as a positive control, gave a clear signal (Figure 3a). In line with this, inhibition of the NF*κ*B pathway by the small-molecule inhibitor SC-514 was not able to abrogate the effect of TRAIL on adipogenesis (Supplementary Figure S1). We thus exclude the NF*κ*B pathway as a mediator of the antiadipogenic effect of TRAIL.

We observed a slight increase in JNK phosphorylation at the beginning of adipogenic differentiation, which did not differ between control and TRAIL-treated cells. This is most likely caused by the change of media upon adipogenic induction. There was no induction of p38 at all, while the positive control displayed a strong signal. Akt was phosphorylated at any investigated time point with no difference between control and TRAIL-treated cells. In contrast, we detected a strong and sustainable phosphorylation of ERK1/2 upon TRAIL treatment starting at 6 h and persisting during the first 24 h of adipogenic differentiation. Densitometric analysis of ERK1/2 phosphorylation is provided in Supplementary Figure S2.

To elucidate if ERK1/2 activation plays a causal role in the antiadipogenic effect of TRAIL, we blocked its phosphorylation by targeting MEK1/2, the specific upstream kinases of ERK1/2, with the small-molecule inhibitor PD98059 (100 *μ*M). This inhibitor blocked both the basal as well as the TRAIL-induced phosphorylation of ERK1/2 (Figure 3b). PD98059 alone had no influence on adipogenic differentiation and it did not modulate the effect of TRAIL on adipogenesis as reflected by comparable differentiation rate and mRNA expression of adipogenic markers genes (Figures 3c and d). Taken together, this set of experiments demonstrates that the antiadipogenic effect of TRAIL is not mediated by the studied non-canonical signaling routes.

### Caspase activation is involved in mediating the antiadipogenic effect of TRAIL

**Figure 4:**
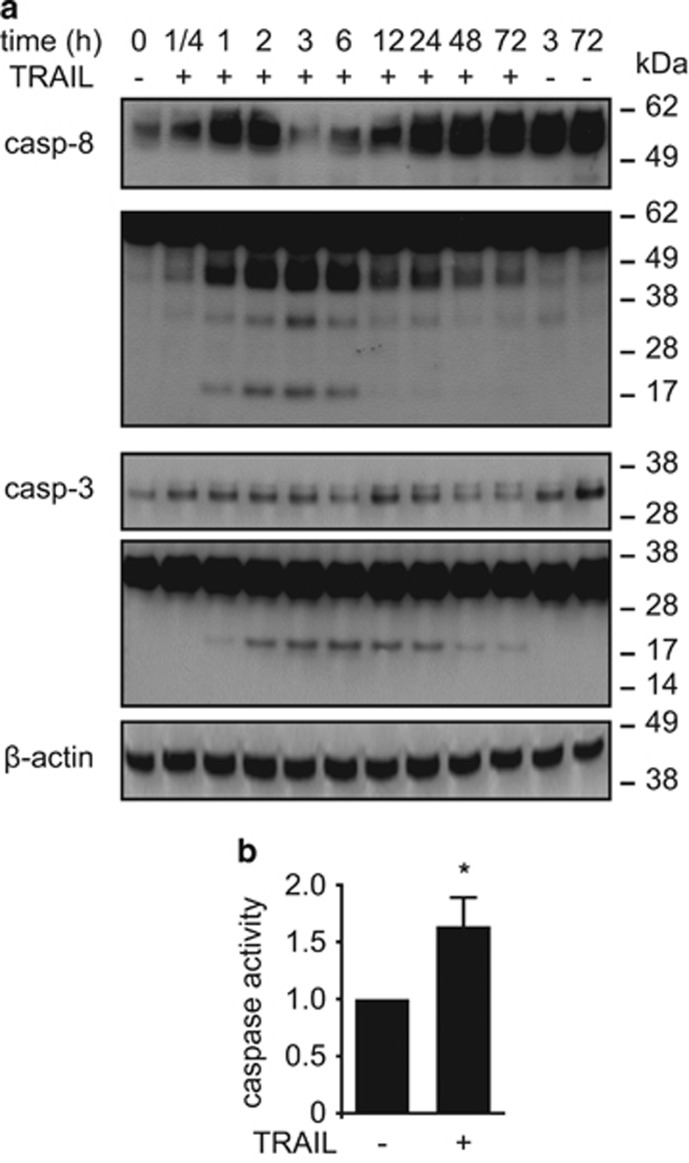
TRAIL induces the cleavage of caspases. (**a**) SGBS cells were treated with TRAIL (30 ng/ml) for different time points (15 min, 1, 2, 6, 12, and 24 h). Protein was isolated and the cleavage of caspase-8 and caspase-3 was analyzed by western blot. *β*-Actin was used as a loading control. The positions of the molecular weight markers (kDa) are indicated. (**b**) SGBS cells were treated with TRAIL (30 ng/ml) for 24 h and the activation of caspases was analyzed with a fluorogenic substrate for activated caspases. Displayed are the means and S.E.M. of five independent experiments. *T*-test was used to test for statistical significance in (**b**). *P<0.05, vehicle *versus* TRAIL

Canonically, TRAIL binds to its surface receptors TRAIL-R1 or TRAIL-R2 leading to receptor trimerization, DISC formation, and cleavage of caspases that can ultimately result in cell death induction.^1^ Under the adipogenic conditions chosen, 30 ng/ml TRAIL triggered a rapid cleavage of caspase-8 with the active p18 fragment clearly detectable from 60 min onwards until 6 h (Figure 4a). Cleavage of caspase-3 occurred within the same timeframe with the active p17 fragment being first visible after 60 min, peaking between 2 and 6 h and weakly persisting for 72 h (Figure 4a). An activity assay using a fluorescently labeled Asp-Glu-Val-Asp (DEVD) peptide that contains a caspase cleavage site revealed that this substrate is cleaved in response to TRAIL treatment (Figure 4b).

**Figure 5:**
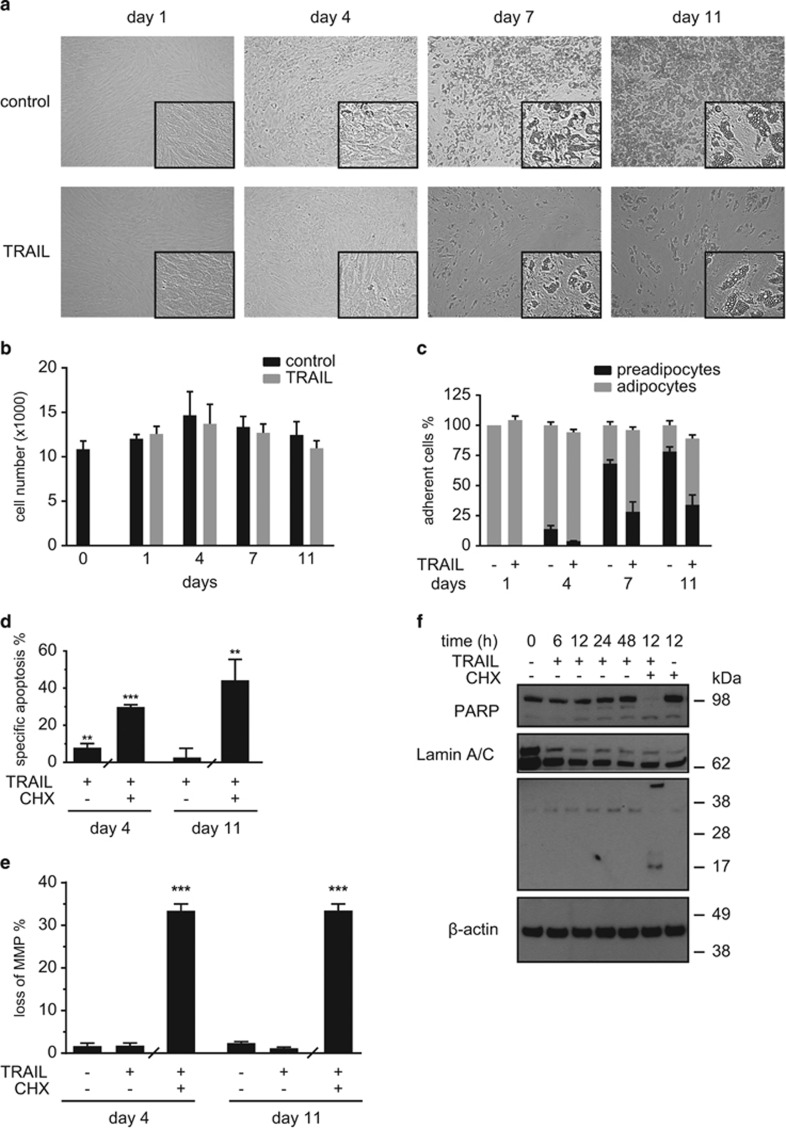
TRAIL induces negligible amounts of apoptosis during adipogenesis of SGBS cells. Human SGBS cells were treated with TRAIL (30 ng/ml) during the first 4 days of adipogenic differentiation. (**a**) Representative photomicrographs of cultures on days 1, 4, 7, and 11 of adipogenesis, magnification × 100. (**b**) The total number of cells and (**c**) the percentages of preadipocytes and adipocytes were determined by cell counting. Displayed are the means and S.E.M. of three independent experiments. (**d** and **e**) Human SGBS cells were treated with TRAIL (30 ng/ml) during the first 4 days of adipogenic differentiation. (**d**) On days 4 and 11 hypodiploid DNA content was measured by flow cytometry and specific apoptosis was calculated. Cells treated with TRAIL in combination with 10 *μ*g/ml CHX for 24 h were used as a positive control. Displayed are the means and S.E.M. of three independent experiments. (**e**) CMXRos staining was performed to detect changes in the mitochondrial membrane potential. Cells treated with TRAIL in combination with 10 *μ*g/ml CHX for 6 h were used as a positive control. Displayed are the means and S.E.M. of three independent experiments. (**f**) SGBS cells were treated with TRAIL (30 ng/ml) for different time points (0, 6, 12, 24, and 48 h). Cells treated with TRAIL in combination with 10 *μ*g/ml CHX for 12 h were used as a positive control. Protein was isolated and the cleavage of PARP and lamin A/C was analyzed by western blot. *β*-Actin was used as a loading control. One representative out of four experiments performed is presented. The positions of the molecular weight markers (kDa) are indicated. One-way ANOVA and Turkey's multiple comparison were used to test for statistical significance in (**b**, **d** and **e**). *P<0.05, **P<0.01,***P<0.001, vehicle *versus* TRAIL and/or CHX

Despite this, there was only negligible apoptosis induction. During the timecourse of adipogenesis, cultures appeared healthy without morphological signs of apoptosis such as loss of plastic adherence (Figure 5a). Likewise, the total adherent cell number was not different between control and TRAIL-treated cells (Figure 5b). However, we observed that the cellular composition of the cultures was altered. On day 7, approximately 68% of the adherent cells were adipocytes and 32% were preadipocytes in the control cultures. In contrast, only 28% of cells were adipocytes and 72% were preadipocytes in TRAIL-treated cultures (Figure 5c).

In line with the morphological observations, two different types of apoptosis assays revealed only low amounts of apoptosis. Measuring hypodiploid DNA content, the percentage of specific apoptosis upon TRAIL treatment was ~8% on day 4 and ~3% on day 11 (Figure 5d). Comparably, the percentage of cells with a decreased mitochondrial membrane potential was ~3% on day 4 and ~1% on day 11 (Figure 5e). As a positive control, we treated adipocytes on day 4 or day 11 with TRAIL in combination with cycloheximide (CHX), which led to the induction of apoptosis in >30% of the cells (Figures 5d and e). In the TRAIL and CHX-treated apoptotic cultures we observed the cleavage of poly(ADP-ribose)-polymerase (PARP) and lamin A/C, whereas this was not seen upon TRAIL treatment alone (Figure 5f).

This set of experiments demonstrates that there is only a weak, negligible induction of apoptosis upon TRAIL treatment under the chosen experimental conditions, which is in agreement with our previous observations that human preadipocytes and adipocytes are resistant to death ligand-induced apoptosis.^16, 17^ We conclude that a reduction in cell numbers is not responsible for the inhibitory effect of TRAIL on adipogenic differentiation.

**Figure 6:**
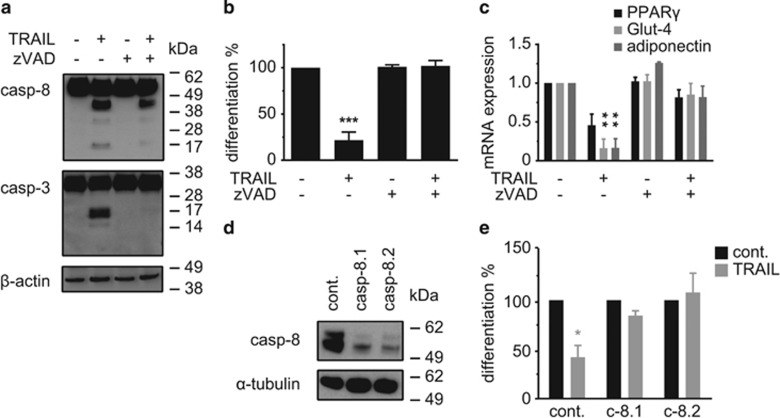
The antiadipogenic effect of TRAIL is mediated by caspases. (**a–c**) SGBS cells were treated with TRAIL (30 ng/ml) in the absence or presence of the pan-caspase inhibitor zVAD.fmk (20 *μ*M). (**a**) Inhibition of caspases by zVAD.fmk was confirmed by western blot after 3 h of treatment. One representative out of three experiments performed is presented. The positions of the molecular weight markers (kDa) are indicated. (**b**) The rate of adipogenic differentiation was determined by cell counting on day 10 of differentiation. Depicted are the means and S.E.M. of three independent experiments. (**c**) RNA was isolated and adipocyte marker gene expression (PPAR*γ*, Glut-4, adiponectin) was determined by qPCR on day 10 of differentiation. The mRNA levels were normalized to the gene HPRT. Displayed are the means and S.E.M. of three independent experiments. (**d** and **e**) SGBS cells were transduced with lentiviruses expressing either a non-targeting shRNA sequence (hyper random sequence, HRS) or shRNA targeting caspase-8 (C8.1 and C8.2) to generate a stable knockdown. (**d**) Knockdown of caspase-8 was controlled by western blot. One representative out of four experiments performed is presented. (**e**) Transduced SGBS cells were treated with TRAIL (10 ng/ml) and the rate of adipogenic differentiation was determined by cell counting on day 10 of differentiation. Displayed is the mean and S.E.M. of three independent experiments. One-way ANOVA and Turkey's multiple comparison were used to test for statistical significance in (**b**, **c** and **e**). *P<0.05; **P<0.01; ***P<0.001, vehicle *versus* TRAIL and/or zVAD

To study if caspase activation is a crucial event in the TRAIL-induced inhibition of adipogenesis, we took advantage of a pan-caspase inhibitor. At a concentration of 20 *μ*M zVAD.fmk completely inhibited the TRAIL-induced processing of both caspase-8 and caspase-3 to their fully active fragments (Figure 6a). While the inhibitor alone had no impact on adipogenesis, it completely abrogated the antiadipogenic effect of TRAIL as reflected by the adipogenic differentiation rate (Figure 6b) and adipocyte marker gene expression (Figure 6c). When zVAD.fmk was added after the initial treatment with TRAIL, for example, 12 h later, it had no impact on the antiadipogenic effect of TRAIL (Supplementary Figure S3). We therefore conclude that the relevant, caspase-dependent step of antiadipogenesis takes place during early adipogenesis.

To further substantiate our findings we decided to perform a genetic knockdown of caspase-8. Using a lentiviral-based shRNA approach we generated SGBS cells with a robust downregulation of caspase-8 expression. Using two different constructs we were able to achieve knockdown efficiencies of ~50% compared with overexpression of a control shRNA with no known target (Figure 6d). While in control cells TRAIL treatment led to a strong and significant inhibition of adipogenic differentiation by 58%, this effect was completely blocked in both caspase-8 knockdown cultures (Figure 6e).

These experiments demonstrate that the antiadipogenic effect of TRAIL requires the presence and the activity of caspase-8.

### TRAIL inhibits the upregulation of early adipogenic transcription factors in a caspase-dependent manner

**Figure 7:**
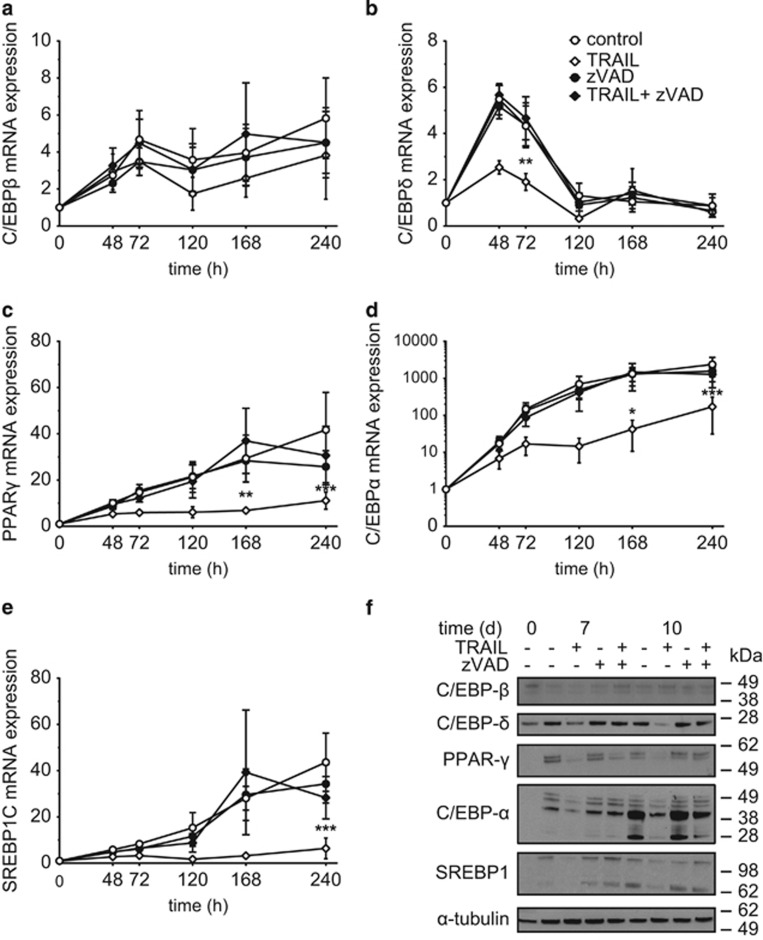
TRAIL reduces the expression of adipogenic transcription factors. SGBS cells were treated with TRAIL (30 ng/ml) in the absence or presence of the pan-caspase inhibitor zVAD.fmk (20 *μ*M) for the first 4 days of adipogenic differentiation. RNA was isolated after 48, 72, 120, 168, and 240 h and qPCR analysis of the adipogenic transcription factors C/EBP*β* (**a**), C/EBP*δ* (**b**), PPAR*γ* (**c**), C/EBP*α* (**d**), and SREBP1C (**e**) was performed. The mRNA levels were normalized to the gene HPRT. Displayed are the means and S.E.M. of three independent experiments. (**f**) Protein was isolated after 7 and 10 days and the protein expression of C/EBP*β*, C/EBP*δ*, PPAR*γ*, C/EBP*α*, and SREBP1C was analyzed by western blot. *α*-Tubulin was used as a loading control. The positions of the molecular weight markers (kDa) are indicated. One representative out of three experiments performed is presented. Two-way ANOVA and Turkey's multiple comparison were used to test for statistical significance in (**a–e**). *P<0.05; **P<0.01; ***P<0.001, vehicle *versus* TRAIL

The adipogenic differentiation program depends on the sequential activation of core transcription factors, including the CCAAT/enhancer binding protein family (C/EBP*α*, *β*, and *δ*), PPAR*γ*, and SREBP-1c.^20^ We therefore analyzed the expression of these factors in the presence and absence of TRAIL. We additionally included a treatment with the caspase inhibitor zVAD.fmk to delineate the involvement of caspase activation. On mRNA level, C/EBP*β* was only weakly induced upon adipogenic stimulation and TRAIL did not exert any significant regulatory effect (Figure 7a). In contrast, TRAIL clearly inhibited the upregulation of C/EBP*δ* (Figure 7b), PPAR*γ* (Figure 7c), C/EBP*α* (Figure 7d), and SREBP-1c (Figure 7e) and this effect was abrogated by co-incubation with zVAD.fmk. Comparable observations were made on protein level (Figure 5f).

These experiments demonstrate that TRAIL inhibits adipogenic differentiation by interfering with the expression of relevant transcription factors in a caspase-dependent manner.

## Discussion

White adipose tissue is characterized by an enormous capability to either shrink or expand. This requires on the one hand tightly equilibrated metabolic processes regulating the volume of existing adipocytes, and on the other hand cellular processes regulating adipocyte number such as precursor cell proliferation, adipogenic differentiation, and cell death. In the present study we identified the death ligand TRAIL as a potent inhibitor of adipogenic differentiation. *Via* TRAIL-R2, TRAIL induced the activation of caspase-8 and -3, leading to reduced expression levels of the early core adipogenic transcription factors C/EBP*α*, C/EBP*δ*, and PPAR*γ*, finally causing an inhibition of terminal differentiation and lipid accumulation.

TRAIL belongs to the TNF superfamiliy^2^ and other family members were already described to have potent antiadipogenic properties. TNF*α* is the most prominent example,^24^ but also CD95L, TWEAK, and LIGHT inhibit adipogenesis.^25, 26, 27^ With this study we add TRAIL to the list of antiadipogenic factors. Interestingly though, TRAIL uses a distinct signaling route to mediate its effects on adipogenesis. While TNF*α* exerts its inhibitory function by NF*κ*B activation,^28^ we did not detect any involvement of this pathway in mediating the effects of TRAIL. Alternatively, TNF*α* is able to block adipogenesis in murine 3T3-L1 cells *via* a *β*-catenin/TCF4 (TCF7L2)-dependent pathway.^29^ However, TRAIL does not seem to signal *via* this route as there was no TRAIL-dependent regulation of canonical Wnt target genes such as c-myc, cyclin D1, or PPAR*δ* (data not shown) as shown for TNF*α* treatment.^29^ Also other TRAIL-activated non-canonical pathways^8^ such as ERK1/2, JNK, and p38 were excluded as players in the antiadipogenic action of TRAIL in human preadipocytes. A TRAIL-induced phosphorylation of ERK1/2 was observed at early time points of adipogenic differentiation. We have shown earlier that TRAIL induces proliferation in human preadipocytes in an ERK1/2-dependent manner.^18^ Therefore, we thought that TRAIL might push the cells into cell cycle and division and by this block the initiation of differentiation. However, cell numbers were not increased in TRAIL-treated cultures (data not shown) and inhibition of ERK1/2 activation with a small-molecule inhibitor of its upstream kinases MEK1/2 did not abrogate the effect of TRAIL, all in all arguing against an involvement of the ERK1/2 pathway in the antiadipogenic effect of TRAIL.

Instead, our data clearly indicate that TRAIL inhibits adipogenic differentiation *via* the canonical, caspase-dependent pathway. This is based on several pieces of evidence. First, TRAIL induced the cleavage and activation of caspase-8 and -3. Second, pharmacological inhibition of caspase activity, and third, genetic knockdown of caspase-8 expression completely abolished the antiadipogenic effect of TRAIL.

Caspases play an important role in differentiation processes in several cell types.^30^ For example, caspase-8 is involved in the differentiation of monocytes to macrophages^31^ and caspase-3 is crucial for the differentiation of mesenchymal stem cells along the osteogenic lineage.^32^ From our data we conclude that the adipogenic differentiation program *per se* does obviously not require the presence or activity of caspases since both the knockdown of caspase-8 as well as chemical inhibition of caspase activity had no effect on the differentiation rate and expression of adipocyte marker genes. They are, however, key players in mediating the antiadipogenic effect of TRAIL. In addition to the induction of cell death, TRAIL has been described as a modulator of differentiation processes in other studies. For example, TRAIL promotes the differentiation of intestinal cells^33^ and induces the maturation of both normal and leukemic myeloid precursor cells to monocytes.^34^ In contrast, TRAIL inhibits human erythropoiesis^35^ as well as the differentiation of osteoclasts.^36^ This underlines that TRAIL exerts its functions in a cell type-specific manner. The remaining question is how preadipocytes are able to survive an activation of caspase-3, which in apoptosis, together with the permeabilization of mitochondria, is often regarded the point of no return, where the destruction of a cell is inevitable.^36^ During the final steps of apoptosis, active caspase-3 is translocated to the nucleus to cleave nuclear substrates, which leads to typical cellular changes such as chromatin condensation and DNA fragmentation.^37, 38^ In adipocytes, active caspase-3 was mainly detected in the cytoplasm after TRAIL treatment.^15^ This spatial distribution might provide one explanation why nuclear integrity is not altered and why there is no execution of cell death despite caspase activation following TRAIL treatment. Instead, other cytoplasmic substrates such as, for example, PPAR*γ* or fatty acid synthase (FASN) are targeted.^15^

The consequence of TRAIL-induced caspase activation is a downregulation of the adipogenic transcription factors C/EBP*α*, C/EBP*δ*, and PPAR*γ* on the mRNA and subsequently also on the protein level. Interestingly, the transcription factor C/EBP*β*, which is induced very early in adipogenic differentiation,^39^ is not regulated by TRAIL on mRNA level, which leads us to conclude that TRAIL mediates its antiadipogenic effect downstream of C/EBP*β* transcription. In line with our observations, TNF*α* treatment of 3T3-L1 cells under adipogenic conditions also leaves C/EBP*β* transcription unaffected, while PPAR*γ* and C/EBP*α* are robustly inhibited.^29^ PPAR*γ* and C/EBP*α* mutually promote their expression^40^ and the transcriptional repression of these factors clearly suffices to inhibit adipogenesis. Both transcription factors are expressed on the mRNA level in the preadipocyte state before the onset of adipogenic differentiation (data not shown). Since caspases exert their functions by the cleavage of substrate proteins, we wondered whether these two transcription factors might be targeted and thereby inactivated. An ExPASy peptide cutter analysis did not predict a cleavage of C/EBP*α* by caspases (data not shown). Likewise, no cleavage was predicted for C/EBP*β* and C/EBP*δ*. In contrast, PPAR*γ* is a known caspase substrate. TNF*α* was shown to induce PPAR*γ* cleavage in murine adipocytes leading to its degradation.^41, 42^ A comparable observation was made by our group. We demonstrated that cleaved PPAR*γ* is retained in the cytoplasm instead of being translocated to the nucleus and therefore not able to induce the transcription of adipocyte-specific genes.^15^ However, in this study, PPAR*γ* protein was first detectable by western blot at 24 h after the induction of adipogenic differentiation and we were unable to detect a cleavage product following TRAIL treatment (data not shown). Using a proteasome inhibitor to prevent the degradation of a possible cleavage product yielded no meaningful insights because the co-treatment with TRAIL resulted in a rapid induction of cell death (data not shown). At this point we can thus not exclude a TRAIL-induced post-transcriptional modification of PPAR*γ* by caspases.

In contrast to humans, mice possess only one death receptor with a functional intracellular domain, DR5, which corresponds to the human TRAIL-R2.^1, 2^ Mice with a systemic knockout of DR5 have no overt adipose tissue phenotype. When maintained on a chow diet, their body weight and fat pad weights are comparable to those of wild-type littermates.^43^ On a diet high in saturated fat, cholesterol, and fructose (FCC), however, knockout animals gain significantly less weight and fat mass and are protected from the development of adipose tissue inflammation, insulin resistance, and hepatic steatosis.^43^ The absence of DR5 might lead to a reduction of apoptosis in adipose tissue and therefore also a decrease in macrophage infiltration and inflammation, which is regarded the initializing event in the pathophysiology of obesity-related co-morbidities.^19^ Alternatively, the phenotype could also be explained by a defect in macrophage function as macrophages derived from knockout animals displayed compromised TRAIL-induced chemotaxis as well as reduced cytokine production.^43^ Furthermore, DR5 might play a direct role in liver cell or adipocyte metabolism. For example, TRAIL was recently shown to induce insulin resistance in human adipocytes, inhibiting insulin-stimulated glucose uptake and lipogenesis.^15^ In contrast to this, the intraperitoneal injection of 10 *μ*g TRAIL once per week resulted in reduced adiposity and improved insulin sensitivity and glucose tolerance in mice on a high-fat diet.^44^ Along the same line, mice lacking TRAIL expression displayed a more severely impaired glucose tolerance and increased systemic inflammation when on a high-fat diet.^45^ These controversial findings led us to conclude that the tissue-specific functions of TRAIL are still far from being understood, but will hopefully be dissected in the future by the use of tissue-specific DR5 knockout animals.

White adipose tissue is highly flexible with a unique capacity to change its volume by manifold. At least in mice both the expression of TRAIL and its receptor DR5 is upregulated in adipose tissue upon high-fat diet.^15, 43^ Acute fasting in mice leads to a downregulation of both ligand and receptor, and their expression is rapidly restored after re-feeding.^15^ This implies that the TRAIL/TRAIL-R system might have an important regulatory role in adipose tissue homeostasis. The finding that TRAIL inhibits adipogenesis and at the same time stimulates proliferation in preadipocytes suggests that it contributes to the maintenance of the pool of adipose tissue precursor cells.^18^ TRAIL also regulates the survival, proliferation, and migration of vascular smooth muscle cells and endothelial cells.^46, 47, 48, 49^ Knowing that the stem cell compartment of adipose tissue resides in perivascular proximity,^50^ it is well conceivable that TRAIL contributes to the preservation of the adipose tissue stem cell niche.

The present study identified TRAIL as a potent antiadipogenic factor in human preadipocytes. TRAIL stimulates the cleavage of caspase-8 and -3, which results in the downregulation of adipogenic transcription factors and decreases lipid accumulation. A decrease in adipogenesis might restrict the expansion of adipose tissue, but likewise increase the risk for ectopic lipid accumulation. TRAIL receptor agonists as well as human recombinant TRAIL are currently tested for their anticancer activity in phase II clinical studies.^9^ Further research is needed to clarify if targeting the TRAIL/TRAIL-R system is a useful strategy to improve or restore adipose tissue function in the context of obesity.

## Materials and Methods

Recombinant human TRAIL was purchased from R&D Systems (#375-TEC; Wiesbaden-Nordenstadt, Germany). Fully human agonistic monoclonal TRAIL-R1 (mapatumumab) and TRAIL-R2 (lexatumumab) antibodies were kind gifts of Human Genome Sciences (Rockville, MD, USA). zVAD.fmk was purchased from Bachem (Bubendorf, Switzerland), SC-514 from Bio-Techne (Wiesbaden-Nordenstadt, Germany), PD98059 from Selleckchem (Houston, TX, USA), and cycloheximide from Sigma-Aldrich (Munich, Germany). Cell culture media and buffers were from Life Technologies (Darmstadt, Germany).

### Subjects and human primary stromal-vascular cell isolation

Primary human stromal-vascular cells were isolated from subcutaneous white adipose tissue by collagenase (Sigma-Aldrich, Munich, Germany) digestion from five female subjects undergoing plastic surgery and cultured as described.^51^ The mean age was 39.2±9.6 years; the mean BMI was 30.0±3.0 kg/m^2^. All procedures were performed according to the Declaration of Helsinki guidelines and authorized by the ethics committee of Ulm University. Written informed consent was obtained from all subjects in advance.

### Cell culture

Human SGBS cells were used as a model system for adipogenesis.^21, 52^ To induce adipogenic differentiation in SGBS and primary human stromal-vascular cells, subconfluent cell cultures were washed with PBS and adipogenic basal medium (serum-free DMEM-F12 (1:1) with 33 *μ*M biotin, 17 *μ*M pantothenate, 20 nM human insulin, 100 nM cortisol, 200 pM triiodothyronine, and 10 *μ*g/ml transferrin) supplemented with 2 *μ*M rosiglitazone, 25 nM dexamethasone, and 250 *μ*M isobutylmethylxanthine was added. TRAIL was added to the cultures for the first 4 days of adipogenesis. After 4 days, the medium was changed to adipogenic basal medium without TRAIL. Analyses were performed 10 days after the induction of adipogenesis. The differentiation rate was determined by counting the number of lipid-laden, differentiated adipocytes (defined by five clearly visible lipid droplets) and undifferentiated cells. Three microscopic fields were counted per well using a net micrometer.

### Triglyceride assay

The cellular triglyceride content of adipocytes was measured using Triglyceride Reagent, Free Glycerol Reagent, and a Glyerol Standard (Sigma-Aldrich, Munich, Germany) according to the manufacturer's instructions.

### RNA isolation and cDNA preparation

Isolation of total RNA was implemented by using the peqGOLD HP total RNA kit (Peqlab, Erlangen, Germany) or the Direct-zol RNA Mini Prep kit (Zymo Research Corporation, Irvine, CA, USA) according to the manufacturers' instructions. cDNA synthesis was performed using SuperScript II Reverse Transcriptase (Life Technologies) according to the manufacturer's instructions.

### Quantitative real-time PCR

qPCR was performed on a LightCycler 2.0 instrument with the LightCycler FastStart DNA Master^PLUS^ SYBR Green I kit (Roche Diagnostics, Mannheim, Germany). The mRNA levels of the genes of interest were first normalized to HPRT (hypoxanthine-guanine-phosphoribosyltransferase, ΔCT value) and then to the respective control condition (ΔΔCT value). The ΔΔCT value was used for the determination of the relative expression. The following oligonucleotide primers were obtained from ThermoScientific (Ulm, Germany): adiponectin forw 5′-GGC CGT GAT GGC AGA GAT-3′, adiponectin rev 5′-CCTTCA GCC CCG GGT ACT-3′ CEBP/*α* forw 5′-GAC CCT CAGCCTTGT TTGTAC TGT ATG CC-3′, CEBP/*α* rev 5′- TTT GGAAAG CTT GTC ATA ACT CCG GTC CC-3′ CEBP/*β* forw 5′- CCG CCCGTG GTG TTA TTT AAA GAA GAAA C GTC-3′, CEBP/*β* rev 5′- GCC CGTAGG AAC ATC TTT AAG CGA TTA CTC AG-3′ CEBP/*δ* forw 5′- CCA TCGACT TCA GCGCCT ACA TCG ACT C- 3′, CEBP/*δ* rev 5′-CCC GCCTTG TGA TTG CTG TTG AAG AGG T-3′ Glut-4 forw 5′-TTC CAACAG ATA GGC TCC GAA G-3′, Glut-4 rev 5′-AAG CAC CGC AGA GAA CAC AG-3′ HPRT forw 5′-GAG ATG GGA GGC CAT CAC ATT GTA GCC CTC-3′, HPRT rev 5′-CTC CAC CAA TTA CTT TTA TGT CCC CTG TTG ACT GGT C-3′ PPAR*γ* forw 5′-GAT CCA GTG GTT GCA GAT TAC AA-3′, PPAR*γ* rev 5′-GAG GGA GTT GGA AGG CTC TTC-3′ SREBP1C forw 5′-TTT CTGACA CGC TTC TTC CTG AGC AGT G-3′, SREBP1C rev 5′-ATG TTCCCG GAATAG CTG AGT CAC CTG G-3′.

### Protein extraction and western blot

Whole-protein extracts were obtained by washing the cells with ice cold PBS, adding lysis buffer (10 mM Tris-HCl pH 7.5, 150 mM NaCl, 2 mM EDTA, 1% Triton X-100, 10% glycerol) supplemented with 1 × cOmplete Proteinase Inhibitor Cocktail and 1 × PhosSTOP Phosphatase Inhibitor Cocktail (Roche Diagnostics), and detaching the cells by scraping. The lysates were incubated for 20 min at 4 °C and then centrifuged at 14 000 r.p.m. for 30 min at 4 °C. Protein concentration was determined using the Pierce BCA Protein Assay kit (Sigma-Aldrich, Munich, Germany). Western blot analysis was performed as described elsewhere.^53^ The following antibodies were used: rabbit anti-phospho Akt, rabbit anti-Akt, mouse anti-phospho ERK1/2, rabbit anti-phospho p38, mouse anti-p38, rabbit anti-phopsho JNK, mouse anti-phospho I*κ*B*α* (S32/S36), rabbit anti-I*κ*B*α*, rabbit anti-caspase-3, rabbit anti-PPAR*γ*, rabbit anti-CEBP/*α*, rabbit anti-PARP (Cell Signaling, Danvers, MA, USA), rabbit anti-CEBP/*β*, rabbit anti-CEBP/*δ* (Santa Cruz Biotechnology, Heidelberg, Germany), mouse anti-JNK, mouse anti-caspase-8 (Alexis, Grünberg, Germany), goat anti-adiponectin, mouse anti-Glut-4 (Bio-Techne), rabbit anti-ERK1/2, mouse anti-*β*-actin (Sigma-Aldrich, Munich, Germany), mouse anti-caspase-8 (Enzo LifeSciences, Lörrach, Germany), mouse anti-*α*-tubulin (Calbiochem/EMD Millipore, Darmstadt, Germany), mouse anti-lamin A/C, and mouse anti-SREBP1 (BD, Franklin Lakes, NJ, USA). HRP-conjugated goat anti-mouse IgG and goat anti-rabbit IgG were from Santa Cruz Biotechnology.

### Lentiviral mediated knockdown of caspase-8

For the knockdown of caspase-8 the BLOCK.iT inducible H1 Lentiviral RNAi System (Life Technologies) was used. All procedures were performed according to the manufacturer's instructions. Two specific shRNA constructs against caspase-8 and a hyper random sequence (HRS) with no complement in the human genome were cloned into the pENTR/H1/TO vector and then transferred into the pLenti4/BLOCK-iT-DEST vector. For the production of lentiviral particles in HEK293FT cells the ViraPower packaging mix and Lipofectamine2000 (Life Technologies) were used. SGBS preadipocytes were transduced with lentiviral supernatant aided by Sequabrene (Sigma-Aldrich, Munich, Germany) and then selected with Zeocin. The following shRNA sequences were used: caspase-8 #1 start 411, 5′-GGA ACA ACT GGACAG TGA AGA-3′, caspase-8 #2 start 693, 5′-GGG TCA TGC TCT ATC AGA TTT-3′, HRS 5′-GAT CAT GTA GATACG CTC A-3′.

### Caspase activity assay

Caspase activity was quantified using CellEvent Caspase-3/7 Green Detection Reagent (Life Technologies). After TRAIL treatment the cells were trypsinized and stained for 30 min at 37 °C with the detection reagent at a final concentration of 2 *μ*M. Cell analysis was performed using a FACSCalibur flow cytometer (BD, Franklin Lakes, USA). For each treatment triplicate wells were prepared.

### Apoptosis assays

Apoptosis was determined by fluorescence-activated cell-sorting (FACSCalibur; Becton Dickinson, Heidelberg, Germany) analysis of DNA fragmentation of propidium iodide-stained nuclei as previously described.^17^ For each treatment triplicate wells were prepared. Specific apoptosis was calculated using the following formula: (observed apoptosis−spontaneous apoptosis) × (100/(100−spontaneous apoptosis)).

The mitochondrial membrane potential was determined as previously described.^17^ For each treatment triplicate wells were prepared. Briefly, CMXRos (Molecular Probes, Karlsruhe, Germany) was added to the cultures at a final concentration of 35 nM. Cells were incubated for 30 min at 37 °C in the presence of the dye and then immediately analyzed by flow cytometry.

### Statistical analysis

All statistical analyses were performed using GraphPad Prism software version 6.01 (La Jolla, CA, USA).

## Abbreviations

BMI: body mass index
CHX: cycloheximide
DISC: death-inducing signaling complex
DR4: death receptor 4
DR5: death receptor 5
FADD: Fas-associated death domain
FLIP: FLICE-like inhibitory protein
HPRT: hypoxanthine-guanine-phosphoribosyltransferase
PARP: poly(ADP-ribose)-polymerase
SGBS: Simpson-Golabi-Behmel syndrome
TNF: tumor necrosis factor
TNFR: TNF receptor
TRAIL: TNF-related apoptosis-inducing ligand
