# Supplementary material for: TRAIL (TNF-related apoptosis-inducing ligand) inhibits human adipocyte differentiation via caspase-mediated downregulation of adipogenic transcription factors
Source: Cell Death Dis. 2016 Oct 13;7(10):e2412–. doi: 10.1038/cddis.2016.286 (PMC5133965; doi:10.1038/cddis.2016.286)
Supplement: Supplementary Appendix [file cddis2016286x1.docx]

**Supplementary Appendix**

**TRAIL (TNF-related apoptosis inducing ligand) inhibits human adipocyte differentiation via caspase-mediated down-regulation of adipogenic transcription factors**

Verena Zoller^1^, Jan-Bernd Funcke^1^, Michaela Keuper^1^, Muad Abd El Hay^1^, Klaus-Michael Debatin^2^, Martin Wabitsch^1^, and Pamela Fischer-Posovszky^1^

**Affiliations:** ^1^ Division of Pediatric Endocrinology and Diabetes, Department of Pediatric and Adolescent Medicine, University Medical Center Ulm, Ulm, Germany; ^2^ Department of Pediatric and Adolescent Medicine, University Medical Center Ulm, Ulm, Germany

**Corresponding author**

Pamela Fischer-Posovszky, PhD

Division of Pediatric Endocrinology and Diabetes

Department of Pediatric and Adolescent Medicine

University Medical Center Ulm

Eythstr. 24

89075 Ulm

Phone: +49 (0)731 500 57415

Email: [pamela.fischer@uniklinik-ulm.de](mailto:pamela.fischer@uniklinik-ulm.de)

**Figure S1**

**
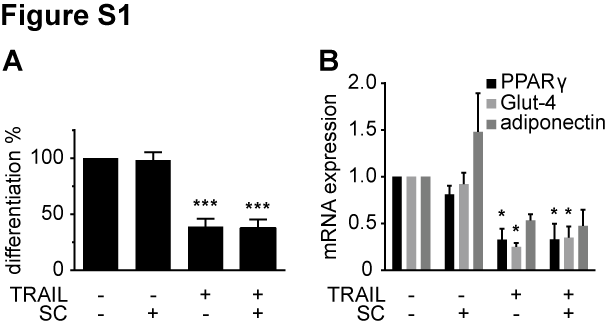
**

**Figure S1: Inhibition of the NFkB pathway via inhibition of IKKβ does not interfere with the anti-adipogenic effect of TRAIL**

**A** Human SGBS cells were treated with 30 ng/ml TRAIL during the first 4 days of adipogenic differentiation in the absence or presence of the IKKb inhibitor SC-514 (100 µM). The rate of adipogenic differentiation was estimated by cell counting at day 10 of differentiation. Displayed are the means and SEM of 3 independent experiments. **B** RNA was isolated and adipocyte marker gene expression (PPAR-γ, GLUT4, adiponectin) was determined by qPCR. The mRNA levels were normalized to the gene HPRT. Displayed are the means and SEM of 3 independent experiments. One-way ANOVA and Turkey’s multiple comparison were used to test for statistical significance *; p0.05, ***; p0.001.

**Figure S2**


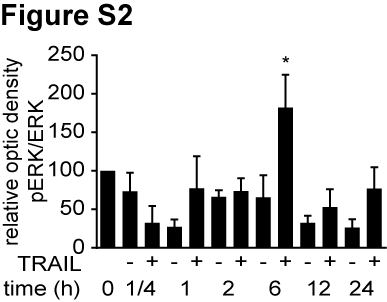


**Figure S2: TRAIL induces the phosphorylation of ERK1/2.**

**A** SGBS cells were treated with TRAIL (30 ng/ml) for different time points (¼ , 1 , 2, 6 , 12, 24 hours) . Protein was isolated and the phosphorylation of ERK1/2 as well as the expression of ERK1/2 were analyzed by western blot analysis.

Relative levels of phosphoERK1/2 and ERK1/2 were analyzed using Image J software. Displayed are the means and SEM of 3 independent experiments. Two-way ANOVA and Sidak´s multiple comparison were used to test for statistical significance. *; p0.05 , vehicle vs. TRAIL.

**Figure S3**

**
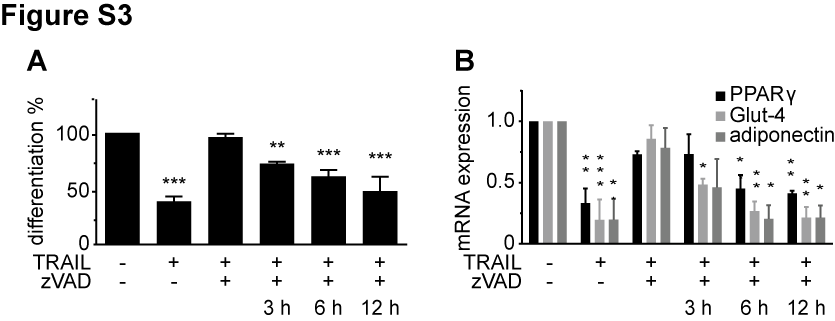
**

**Figure S3: The anti-adipogenic effect of TRAIL is mediated by caspases**

SGBS cells were treated with TRAIL (30 ng/ml) for the first four days of adipogenic differentiation. The pan-caspase inhibitor zVAD.fmk (20 µM) was added either together with TRAIL at the start of adipogenesis or after 3, 6, or 12 hours. **A** The rate of adipogenic differentiation was estimated by cell counting at day 10 of differentiation. Displayed are the means and SEM of 3 independent experiments. **B** RNA was isolated and adipocyte marker gene expression (PPAR-γ, GLUT4, adiponectin) was determined by qPCR. The mRNA levels were normalized to the gene HPRT. Displayed are the means and SEM of 3 independent experiments. One-way ANOVA and Turkey´s multiple comparison were used to test for statistical significance *; p0.05, ***; p0.001.
